# Perceived readiness of South African community service speech–language therapists to manage complex paediatric dysphagia

**DOI:** 10.4102/sajcd.v72i2.1110

**Published:** 2025-11-22

**Authors:** Lavanya Naidoo, Cynthia Sawasawa, Nabeela Desai

**Affiliations:** 1 Department of Speech-Pathology and Audiology, Faculty of Humanities, University of the Witwatersrand, Johannesburg, South Africa

**Keywords:** swallowing, South Africa, community service, speech–language therapists, paediatric, feeding and swallowing difficulties

## Abstract

**Background:**

Feeding and swallowing difficulties usually demand specialised knowledge and skills for effective assessment and management. In South Africa, community service speech–language therapists (SLTs) play an integral role in delivering care within the public healthcare system, where the ratio of patients to medical professionals is notably disproportionate. However, the extent to which they feel prepared when managing these conditions remains relatively underexplored.

**Objectives:**

To explore the extent to which community service SLTs feel prepared when managing paediatric feeding and swallowing difficulties, the challenges they experience, and the resources and support systems available to them.

**Method:**

An explorative descriptive qualitative design was used in this study. Data were collected using online surveys and analysed using inductive thematic analysis. Twelve clinicians participated in this study, all of whom worked in different public healthcare institutions throughout South Africa.

**Results:**

Three themes emerged: (1) lack of confidence and self-doubt in their clinical skills; (2) systemic factors within the public health sector that impede service delivery; and (3) difficulty in navigating caregiver health beliefs and the duty of care.

**Conclusion:**

This study provides a nuanced understanding of the lack of preparedness community service SLTs feel when managing children with complex feeding and swallowing difficulties. The findings further underscore the need for additional mentorship support for these therapists, especially in facilities where there are no established speech therapy services.

**Contribution:**

The study has implications for improved service delivery initiatives and curriculum reform at institutions of higher learning.

## Introduction

The assessment and management of feeding and swallowing difficulties in children with complex medical needs can pose a considerable challenge for novice healthcare professionals (Lee et al., 2024). These difficulties may arise from various medical conditions associated with feeding and swallowing difficulties, such as neurological disorders (Calderone et al., 2025), genetic syndromes (Cooper-Brown et al., 2008) and congenital abnormalities (Dharmaraj et al., 2023), and for all of which these children will frequently require highly specialised care and intervention (Printza et al., 2022). In South Africa, a country facing significant service delivery challenges, managing feeding and swallowing difficulties is often difficult because of limited resources, unequal access to healthcare services and varying levels of expertise (Maphumulo & Bhengu, 2019). When assessing and managing children with feeding and swallowing difficulties, various healthcare professionals collaborate to address the complex needs of the child, and to ensure their continued and comprehensive care (Taberna et al., 2020). Speech–language therapists (SLTs) play an integral role in assessing, diagnosing and managing feeding and swallowing disorders in children, to meet their complex medical needs (Duffy, 2018), although doing so frequently demands specialised skills and experience.

Despite their undergraduate training in paediatric dysphagia, many newly qualified SLTs report feeling underprepared and lacking confidence to manage these complex cases effectively (Caesar & Kitila, 2020; Zimmerman, 2016). This gap between formal education and clinical readiness has been attributed to limited hands-on training opportunities, insufficient exposure to complex cases during training, and a lack of ongoing mentorship in early clinical practice. In South Africa, these challenges are further compounded by systemic factors such as resource constraints, high staff turnover and an uneven distribution of permanent SLTs in public healthcare (Mbola et al., 2018; Njilo & Ross, 2025). Additionally, owing to the shortage of permanently employed staff in different public healthcare institutions, the responsibility for ensuring service provision often falls to community service SLTs (Mbola et al., 2018).

In South Africa, community service is a compulsory year of practice for newly qualified healthcare professionals, including SLTs, designed to address service inequities in under-resourced areas (Reid et al., 2018; Singh et al., 2015). This period typically represents the SLTs’ first experience of independent clinical practice, with placements ranging from urban hospitals to rural clinics. Rural, urban and peri-urban areas here are distinguished by population density, infrastructure development and proximity to economic centres (Rajendran et al., 2024). Rural areas are typically characterised by low population density, limited access to healthcare and educational services, underdeveloped infrastructure and significant travel distances to reach basic services (Nkosi, 2024). In contrast, urban areas are defined by high population density, greater access to resources and more developed infrastructure, including hospitals, schools and public transport (Turok & Borel-Saladin, 2014). Peri-urban areas exist at the interface between rural and urban zones, and they often experience rapid population growth and inconsistent access to healthcare services (Mthiyane et al., 2022). Understanding these distinctions is crucial when examining the placement contexts of community service SLTs, as each setting presents distinct resource constraints, service demands and contextual challenges. As a result, community service SLTs are frequently placed in settings where they are expected to manage complex caseloads and navigate systemic challenges with minimal supervision or institutional support (Singh et al., 2015). While supervision is not a community service requirement, the inference is that the inclusion of a supervisor and/or mentor can be a useful resource for these developing clinicians (Mupawose et al., 2021). Although this service year plays a vital role in healthcare delivery, the readiness of SLTs to meet these demands remains under-researched, highlighting a significant gap in the literature.

Existing studies suggested that while community service provides valuable exposure, many graduates feel underprepared for autonomous decision-making, especially in under-resourced environments (Pillay & Kathard, 2015). This sense of unpreparedness is particularly acute when managing dysphagia, a high-risk, skill-intensive area of practice (Coutts, 2019), and even more so in paediatric contexts, where clinical reasoning, caregiver communication and multidisciplinary collaboration are essential. Without adequate training, mentorship or contextual support, SLTs often experience a difficult transition from university to clinical practice, particularly in handling the clinical and ethical complexities of paediatric dysphagia in community settings.

### Background and rationale

Feeding and swallowing difficulties often receive limited attention within the South African public healthcare sector (Kater, 2022). The prevailing focus, within the sector being on the quadruple burden of disease (Achoki et al., 2022), means that health problems like feeding and swallowing difficulties are frequently overlooked in policy development, training and service delivery efforts. Notably, there is also a marked lack of continued professional development (CPD) opportunities targeting the assessment and management of complex feeding disorders, leaving SLTs underprepared, especially in the face of complicated clinical presentations.

Globally, SLTs report feeling underprepared when managing paediatric dysphagia (Caesar & Kitila, 2020; Zimmerman, 2016). Many South African healthcare facilities often depend on community service therapists to manage these high-risk feeding cases, often without adequate clinical exposure, supervision or guidance, and/or access to additional training (Msomi & Ross, 2024; Singh et al., 2015). The absence of structured support and/or relevant training, available to community service SLTs, not only places these therapists in ethically challenging situations but also compromises the quality and safety of care delivered to medically vulnerable children (Ong et al., 2023). The perceived lack of readiness also relates to broader issues such as the sudden transition from structured academic settings to under-resourced and high-pressure public healthcare environments.

Newly qualified professionals, including SLTs, are assigned to work in areas of need across the country (Singh et al., 2015). For many SLTs, the complexity of cases encountered during community service can vary significantly depending on the healthcare facility where they are placed. The expectations placed on these novice professionals may be overwhelming, especially when dealing with high-need cases such as children with complex medical conditions (Coutts, 2019). Therefore, it is essential to understand how community service SLTs inherently perceive their level of readiness to manage paediatric feeding and swallowing difficulties. This information can be used to eventually guide focused training, supervision, and support plans that will better prepare these professionals at a pivotal point in their careers and enhance the quality of care provided to vulnerable paediatric populations.

Understanding perceived readiness involves exploring multiple dimensions, including SLTs’ self-assessment of their skills, their ability to recognise and address paediatric feeding and swallowing disorders, and their perceived level of preparedness to engage in interventions for children with complex medical conditions. Furthermore, research has also considered the broader socio-economic and healthcare context within which community service SLTs operate (Khoza-Shangase, 2025). South Africa’s healthcare system is marked by significant disparities between urban and rural areas, with community service SLTs often working in rural or underserved settings where access to specialist training and resources may be limited (Ngene et al., 2023). These disparities may influence the perceived readiness of therapists and may highlight the need for more comprehensive support and development programmes, particularly for those working in resource-constrained environments.

By identifying the challenges faced by community service SLTs in managing complex paediatric feeding and swallowing cases, this study aims to provide valuable insights into how the training, mentorship and resources available to early-career therapists can be enhanced. Ultimately, the goal is to improve the quality of care provided to children with complex medical needs in South Africa, ensuring they receive the necessary interventions to address feeding and swallowing difficulties and improve their overall health outcomes.

### Aims and objectives

This study aims to investigate how prepared community service SLTs feel when managing children diagnosed with complex medical conditions who experience feeding and swallowing difficulties. The following objectives informed the study’s aim:

- To investigate the extent to which community service SLTs feel prepared when managing feeding and swallowing issues in children with complex medical conditions.
- To explore the challenges encountered by community service SLTs when managing children with complex medical conditions who have feeding and swallowing difficulties in South Africa.
- To identify the resources and support systems available to South African community service SLTs when managing the feeding and swallowing difficulties of children with complex medical conditions.

## Research methods and design

### Research design

A qualitative descriptive research design was used in this explorative study. A qualitative research approach guided by a descriptive design provides in-depth insights into real-world problems by enabling empirical exploration of contemporary issues through the participants’ lived experiences (Moser & Korstjens, 2017; Muzari et al., 2022). Given the contextual, emotional and largely subjective reality that accompanies clinical practice for community service SLTs, it was essential to record the participants’ lived opinions, perceptions and reflections in a naturalistic and minimally interpretive manner. In contrast to quantitative methods or more interpretive qualitative approaches, which may overlook or oversimplify (Wiesner, 2022), this design provides a rich, nuanced understanding of how newly qualified SLTs understand, experience and navigate their clinical roles.

### Recruitment strategy

A purposive, convenience-based recruitment strategy was used to recruit participants (Etikan et al., 2016). Community service SLTs were invited to participate in the study by clicking the survey link on the research poster, which was designed and shared in a WhatsApp group by the relevant group administrator. A WhatsApp group made up exclusively of community service SLTs was purposively selected for this study, as it consisted entirely of the target population and represented a diverse range of participants from various geographical regions across South Africa. Interested participants were subsequently provided with a participant information sheet outlining the study’s purpose, procedures, risks and benefits. Participants were also asked to provide their consent electronically by selecting the relevant option before being directed to the survey. Participants were then contacted by N.D., *via* telephone and/or email so that the study poster and survey link could be forwarded to them to access independently.

### Participant description

Participants were selected based on the study’s inclusion criteria (Palinkas et al., 2016), namely that they were currently registered and employed as a community service SLT in 2024 and were actively managing children with complex feeding and swallowing difficulties. The sample size for this study was 12 community service SLTs working with children diagnosed with medically complex conditions, as this was sufficient to achieve data saturation (Clarke & Braun, 2013). This sample size was chosen based on the fact that data sufficiency, depth of understanding, rigour and credibility of findings will be achieved (Hennink & Kaiser, 2022).

### Data collection

#### Data collection tool

An electronic, self-administered survey was developed (in Google Forms) and used to obtain data concerning the participants. The survey was designed in alignment with the study’s objectives and underwent a peer review process to enhance clarity, relevance and content validity prior to distribution. The following participant information was obtained: (1) demographics, including the university they previously attended, the public healthcare institution where they are currently employed, as well as the percentage of children, they manage, who are diagnosed with complex medical conditions. The survey further aimed to source data concerning the participants’ (2) perceptions of their preparedness when providing management services to these children; (3) the specific training and/or education participants received related to this population group, and whether or not they found this training beneficial in their current role; (4) intrinsic concerns related to the management of these children at the onset of their employment; and (5) extrinsic factors related to challenges the participants faced in their respective contexts that impacted their ability to manage these children effectively. The survey also required participants to (6) reflect on cultural and/or societal challenges they have experienced in their respective contexts. The survey concluded by requesting participants to comment on (7) the resources and/or support systems available to them to help manage their clients effectively as well as the availability of these resources.

The survey was reviewed by SLTs with clinical expertise and/or who have conducted postgraduate research in the area of paediatric dysphagia. Feedback was gathered through written comments. The survey was subsequently revised to address suggestions related to terminology, question structure and the inclusion of context-specific examples. The reliability of the questionnaire was supported through careful attention to the consistency of its structure, wording and administration (Ranganathan & Caduff, 2023). To enhance the quality and depth of the data collected, the survey was designed to include a combination of closed-ended and open-ended questions. Closed-ended items featured dichotomous options as well as both positively and negatively worded statements, which helped minimise response bias and encouraged thoughtful engagement with the content (Hyman & Sierra, 2016). Open-ended questions were included to provide participants with opportunities to elaborate on their responses, allowing for a better understanding of their lived experiences (Hyman & Sierra, 2016). This design facilitated the identification of recurring themes across participants and contributed to the overall trustworthiness and credibility of the findings.

Out of the 106 community service SLTs approached, 12 therapists participated in the study and represented a broad spectrum of accredited higher education training institutions throughout South Africa. All the participants (*n* = 12; 100%) were employed as community service SLTs at a public healthcare institution, two of whom worked at a primary healthcare clinic (17%), four worked at a district hospital (33%), two worked at regional hospitals (17%) and four of whom (33%) worked at tertiary hospitals. The participants in the study varied in terms of their undergraduate education and current employment, representing rural, peri-urban and urban facilities across South Africa. All participants reported having had experience managing a child diagnosed with a complex medical condition and who subsequently presented with a feeding and swallowing difficulty; the total percentage of children seen varied (10% – 75%) among the participants. Table 1 illustrates the participants’ demographics.

**TABLE 1:** Participants’ demographics.

Participant	University	Site	Province of employment during community service year	Percentage of children with complex medical conditions managed
1	Stellenbosch University	District hospital	KwaZulu-Natal	10
2	University of Pretoria	Tertiary hospital	Gauteng	25
3	University of Pretoria	Regional hospital	Gauteng	75
4	University of Pretoria	Primary healthcare clinic	Free State	10
5	Stellenbosch University	District hospital	Northern Cape	15–20
6	University of the Witwatersrand	Tertiary hospital	Mpumalanga	25
7	University of the Witwatersrand	Regional hospital	Gauteng	50
8	Stellenbosch University	Primary healthcare clinic	Western Cape	10
9	University of KwaZulu-Natal	Tertiary hospital	KwaZulu-Natal	50
10	Stellenbosch University	District hospital	Gauteng	10
11	University of Cape Town	Tertiary hospital	Gauteng	75
12	University of the Witwatersrand	District hospital	North West	25

### Data analysis

Data from the qualitative questions were analysed using inductive thematic analysis. This approach allowed for themes to emerge directly from the data, rather than being shaped by preconceived categories, thereby ensuring that the analysis remained grounded in the participants’ own words and perspectives (Naeem et al., 2023). This process supported the generation of rich, nuanced insights while maintaining the integrity of participants’ lived experiences (Zelčāne & Pipere, 2023). This data were used together with the descriptive statistics automatically generated from the Google Forms for the demographic questions. The generated data were thoroughly read to establish familiarity with the content and context. This process proved particularly helpful when developing an understanding of the dataset and identifying emerging themes from the dataset. Once emerging themes became apparent, L.N. began assigning labels or codes to the data to establish meaningful connections aligned with the research objectives. After initial coding, overarching themes emerged and similar codes were subsequently organised and/or clustered into broader themes by L.N., C.S. and N.D. The identified themes were reviewed and analysed by the authors using visual representations, such as mind maps to deepen the understanding of the dataset and ensure the thematic analysis was coherent, comprehensive and reflective of its complexity. Frequent member checks among the authors were conducted to ensure objectivity and consensus regarding the emergent themes.

### Trustworthiness

Ensuring the trustworthiness of qualitative research is crucial to producing reliable and meaningful findings. This study adopted strategies to enhance the credibility, transferability, dependability and confirmability of the data, in line with the guidelines outlined by Elo et al. (2014) and Shenton (2004). The following measures were taken to ensure the rigour and trustworthiness of the study’s findings.

*Credibility* was established through peer debriefing to minimise potential biases. This process involved regular discussions between the authors. The authors were able to refine emerging themes, ensure that findings were firmly grounded in the data, and thus enhance the transparency and trustworthiness of the analysis (Nowell et al., 2017). *Transferability* was supported through the inclusion of detailed clinician demographic questions to provide contextual depth and through purposive sampling to ensure participants had direct experience with the phenomenon under investigation (Campbell et al., 2020). This intentional selection enabled the researchers to gather rich, relevant insights, enhancing the potential applicability of the findings to similar settings or population groups. *Dependability* was supported by maintaining a thorough record of the study’s methods and design, and *confirmability* was achieved through regular auditing between the authors to mitigate bias.

### Ethical considerations

Ethical approval was granted by the University of the Witwatersrand’s Human Research Ethics Committee (Non-Medical) (reference number: STA_2024_44). Gatekeeper permission was obtained from the WhatsApp group administrator prior to the study advert being posted on the group. Also, a written consent was obtained prior to the initiation of the data collection process.

## Results

The results of this study indicate that both intrinsic and extrinsic factors influence community service SLTs’ perceived readiness to manage feeding and swallowing difficulties in children diagnosed with complex medical conditions. There are three main themes identified in this study, namely: (1) the lack of confidence and self-doubt in their clinical skills; (2) systemic factors within the public health sector that impede service delivery; and (3) difficulty in navigating caregiver health beliefs and the duty of care.

### Theme 1: Lack of confidence and self-doubt in their clinical skills

While some participants felt confident in their ability to manage paediatric dysphagia difficulties, particularly in routine or less complex cases, others described a lack of confidence and, at times, confusion when treating complex cases. In the quote below, one participant indicated that, in general, she feels prepared and confident to manage dysphagia patients, but complex cases bring about confusion:

‘I now feel prepared and quite confident in my ability to manage a case with dysphagia from the beginning until a patient is discharged. However, there are still cases which sometimes leave me puzzled.’ (P3, community service SLT, female)

The complexity of treating patients with multiple comorbidities, such as cerebral palsy and epilepsy, led to uncertainty about treatment options and the decision-making process surrounding treatment options:

‘Those with cerebral palsy plus epilepsy plus heart issues are very complex and all different – I do not know when to allow soft feeds or not, it is difficult.’ (P1, community service SLT, female)

‘I was concerned about my actual management of my patients and would question all my decisions due to the complex medical history. There are certain diagnoses that put patients at risk for certain things, therefore, I was concerned about the accuracy of patient care [*that*] I was providing …’ (P11, community service SLT, female)

Other participants reported that the lack of supervision, namely, senior SLTs, who they could consult when treating complex cases, increased their anxiety, especially when they were required to independently make clinical decisions:

‘I think the biggest hurdle is seeing a patient with no supervision and no input from other therapists. During my undergraduate, I was always accompanied either by another student or by a clinical supervisor. It’s very high stakes to be the sole person making the call.’ (P10, community service SLT, female)

‘My main concerns were probably with regards to not having a Speech Therapy supervisor for guidance … as well as being in under-resourced areas.’ (P4, community service SLT, female)

Participants also reported significant gaps in both their undergraduate training and clinical exposure to paediatric dysphagia. While others found themselves uncertain when managing complex cases because of limited hands-on experience:

‘I didn’t have much exposure … I could identify issues but couldn’t treat them.’ (P9, community service SLT, female)

‘My lack of experience often leaves me doubting myself.’ (P10, community service SLT, female)

### Theme 2: Systemic factors within the public health sector that impede efficient paediatric dysphagia service delivery

This theme highlights how systemic factors undermine the confidence of community service SLTs in delivering effective paediatric dysphagia management. Although SLTs may have foundational knowledge, the broader public health context often restricts their ability to apply this confidently and consistently. These structural constraints not only impede service delivery but also contribute to uncertainty and professional insecurity, particularly when handling complex feeding and swallowing cases in children.

Participants indicated that a lack of resources within healthcare facilities was a significant barrier to effective treatment. Participants reported having challenges accessing thickening agents, limited access to varying consistencies and food textures that they need when conducting therapy, and specialised feeding equipment, all of which affected the overall quality of care:

‘We don’t always have specific foods and consistencies available in my hospital. We have also been unable to acquire specialized feeding equipment or thickener.’ (P1, community service SLT, female)

‘The lack of support for SLT services is evident through understaffing and resources. Often times my professional opinion is not valued or taken into account when managing a patient.’ (P6, community service SLT, female)

The lack of objective assessments was a key challenge in managing feeding and swallowing difficulties, which therefore required participants to rely solely on their limited clinical experience. The reliance on subjective clinical decisions often resulted in reduced confidence in the decisions that they had made about their treatment strategies:

‘No objective assessments means my findings are solely based on clinical expertise and/or opinion. A lot of the times, these patients have fluctuations in their feeding, so having one assessment session [*to*] determine their management plan is not ideal.’ (P10, community service SLT, female)

‘It is difficult to get a detailed idea of what is happening on an anatomical level with some of the children with complex feeding disorders. I feel less confident in my management plans in these cases.’ (P4, community service SLT, female)

A recurring theme among participants is also the lack of awareness regarding their role and the importance of Speech–Language Therapy within the multidisciplinary team (MDT). This gap in understanding often resulted in inappropriate or delayed referrals, compromising patient outcomes. As one participant noted:

‘Lack of awareness about our role means that patients get referred later than would be ideal, and some patients may not be referred for the appropriate intervention.’ (P12, community service SLT, female)

‘MDT [*multidisciplinary team*] cooperation is difficult due to high caseloads and limited time. Doctors discharge without SLT approval.’ (P11, community service SLT, female)

Participants reported that nurses often do not adhere to feeding recommendations, which may have negative implications on a child’s health and their overall progress, thus potentially increasing the length of stay in the hospital.

‘Occasionally, with nursing staff not following through with feeding suggestions, pts [*patients*] may aspirate or lose weight as there is increased spillage or vomiting, resulting in inadequate nutritional intake.’ (P3, community service SLT, female)

‘There is little carryover with nurses when food modification is made, so the children end up having to eat consistencies they cannot tolerate.’ (P7, community service SLT, female)

### Theme 3: Difficulty navigating caregiver health beliefs, language barriers, socio-economic challenges and the duty of care

Several participants noted resistance from caregivers, particularly concerning medical interventions, such as the insertion of a percutaneous endoscopic gastrostomy (PEG), which conflicted with traditional beliefs:

‘A handful of my pts who required PEGs, there was resistance from the parents. This was either due to cultural beliefs or societal embarrassment.’ (P3, community service SLT, female)

Some participants reported that they wanted to consult with a traditional healer before continuing with medical interventions, which was difficult for the SLTs to understand:

‘Yes. I once had a patient with a very complex medical history and required a PEG, but the parents refused as a traditional healer had requested them not to perform any medical surgeries before being seen by the traditional healer.’ (P11, community service SLT, female)

‘Patients refusing PEG [*percutaneous endoscopic gastrostomy*] or NGT [*nasogastric tube*] based on beliefs.’ (P1, community service SLT, female)

Language barriers emerged as a critical challenge in both communication with caregivers and collaboration with healthcare professionals, which directly impacted the rapport between them, the implementation of feeding interventions, and at times led to miscommunication:

‘The language barrier makes carryover of strategies difficult.’ (P12, community service SLT, female)

‘Patients who are isiZulu speaking have to wait for my words to be translated as I am English speaking and learning isiZulu. A patient once thought I had insulted her child as she could not understand my words yet. However, besides this I have not had any other challenges. Being respectful and kind to your patients and their caregivers is a must, regardless of their and your society and culture.’ (P9, community service SLT, female)

Socio-economic status emerged as a major barrier to effective feeding therapy because some caregivers found it challenging to implement strategies for their children who require texture-modified diets. This is because many caregivers experience financial challenges and food insecurity:

‘Poverty definitely plays a role. Parents can’t afford transport to access healthcare as often as needed, or may not be able to afford specific dietary requirements their child may have. There is also a big belief in traditional medicine which often clashes with our treatment.’ (P2, community service SLT, female)

‘Just food and/or resource insecurity – for example, when wanting to recommend patients to blend their food to make a smooth puree but they don’t have a blender and/or recommending pumpkin or potato but the patients cannot afford it. I then have to collaborate with the dietician on affordable foods with the correct consistency for the patient.’ (P5, community service SLT, female)

## Discussion

The overall findings of this study indicate that community service SLTs lack confidence and feel underprepared to manage feeding and swallowing difficulties in paediatric patients with complex medical conditions. While this lack of confidence is most directly reflected in theme 1, themes 2 and 3 further illuminate the external and interpersonal challenges that exacerbate this sense of under-preparedness. Participants described insufficient senior mentorship, poorly resourced facilities, and communication and cultural barriers as key factors negatively impacting both their service delivery and professional confidence. Systemic issues, such as resource shortages, limited supervision, heavy caseloads and fragmented interdisciplinary collaboration, combined with the complexities of navigating caregiver beliefs, language differences, socio-economic challenges and ethical responsibilities, collectively hinder the SLTs’ ability to provide effective care and further undermine their confidence in managing complex paediatric dysphagia cases.

Participants in the study indicated that they lack confidence and are doubtful about their clinical skills when treating complex cases. Their perceptions were caused by their limited clinical exposure during undergraduate clinical training and the lack of senior SLTs that they could consult when faced with challenging cases. Singh et al. (2015) found that community service therapists in South Africa attributed their lack of confidence to limited clinical learning opportunities, with some universities offering minimal or no clinical training in this area. Similarly, SLTs in the United States have reported feeling underprepared to work with paediatric patients with feeding difficulties immediately after academic training, citing unmet educational needs (Thompson et al., 2024). These findings suggest that there is a need to improve undergraduate training by increasing clinical learning opportunities, especially with complex paediatric cases. This is because there is a direct link between clinical exposure and preparedness in clinical settings where independent practice is a requirement, especially in contexts, such as South Africa, where there are limited human resources (Pillay et al., 2020).

However, while the overall findings of this study suggest that many community service SLTs report feeling underprepared and lacking confidence in managing paediatric feeding and swallowing difficulties, particularly in medically complex cases, this experience is not universal. Some participants expressed a sense of preparedness for general dysphagia management, identifying challenges primarily when faced with complex or unfamiliar medical conditions. This reflects a natural aspect of clinical skill development, where confidence builds progressively with case exposure and practical experience. However, the transition from academic training to independent practice can be particularly demanding in the South African public health context, where systemic and contextual challenges intensify the learning curve.

The absence of structured mentoring emerged as a consistent challenge, with studies such as Kamal et al. (2012) highlighting that Malaysian-trained SLTs often manage dysphagia cases independently without prior mentorship. Coutts (2019) similarly found that mentorship during community service can enhance clinical skills, reduce risks of adverse patient outcomes and support professional development through learning from experience and fostering critical thinking. While focused on adult dysphagia, these findings underscore the broader value of mentorship across contexts. To align with the goals of community-based rehabilitation, mentorship should be re-conceptualised not only as a support for less experienced therapists but also as a means of empowering all clinicians working in resource-limited settings. This includes expanding access to CPD activities, seeking mentorship beyond the SLP profession when necessary and strengthening multidisciplinary team collaboration. Such an approach addresses professional isolation and enhances the capacity of therapists to deliver safe, effective and contextually relevant care.

The lack of resources within the public health sector as further articulated by participants in this study is one that resonates across many health disciplines in South Africa (Malakoane et al., 2020; Maphumulo & Bhengu, 2019). This may justify the challenges that community service SLTs experienced with carry-over of therapy recommendations because of the fact that nurses are understaffed and overworked. In addition to the disproportionate ratio of SLTs to patients (Pillay et al., 2020), the current public health system is facing challenges such as shortages of resources and equipment. Maphumulo and Bhengu (2019) described shortages of surgical equipment and diagnostic scans, both of which have detrimental effects on health outcomes. However, there is limited literature describing the lack of access of videofluoroscopic machines in the public health sector, and how this impacts the health outcomes and the services provided by SLTs in South Africa (Coutts & Pillay, 2021).

The participants in this study described challenging experiences when working with linguistically and culturally diverse caregivers. The linguistic differences impact on the participant’s information-giving practice, which in turn affected the caregiver’s ability to implement therapy recommendations. Similarly, Harrison et al. (2020) reported that language barriers result in miscommunication and missed information-giving opportunities, thereby negatively impacting the quality of services provided to patients. Additionally, Maul (2015) found that SLTs find it challenging to provide services when there are language differences between the SLT and families, which further impacts their ability to communicate therapy goals and progress. Participants in the current study also reported that they experienced challenges navigating cultural differences regarding health beliefs, especially the belief in traditional medicine. Medical pluralism is a common practice in South Africa (Galvin et al., 2023); however, in healthcare settings, healthcare professionals ascribe to a biomedical Western practice that often conflicts with cultural and traditional health practices. Masso et al. (2025) found that cultural differences cause mismatches between SLT and family expectations of management and the decision-making process.

The impact of low caregiver socio-economic statuses on the carry-over of dysphagia therapy was also highlighted by the participants in this study. Despite the increased access to child-support grants, which are provided by the South African government, many families still live in poverty (Zembe-Mkabile et al., 2015). Speech–language therapists reported similar experiences in a study conducted by Maul (2015), when they indicated that socio-economic factors often impact caregiver adherence to therapy because of challenges such as limited access to transportation. It is therefore important that SLTs consider socio-economic factors and resources accessible to caregivers when developing therapy recommendations, which in turn affects the effectiveness of the dysphagia management implemented. This highlights the importance of SLTs being prepared and confident in considering these socio-economic challenges and available resources when developing realistic and feasible dysphagia therapy recommendations for paediatric patients.

In light of the challenges identified, it is essential to explore multiple strategies to enhance SLTs’ clinical preparedness for managing paediatric dysphagia. While simulated learning has been increasingly recognised as an effective pedagogical tool, its structured integration into undergraduate training can provide students with realistic, safe opportunities to develop clinical reasoning and decision-making skills before engaging in high-stakes environments (Elendu et al., 2024). Simulations can replicate complex paediatric feeding scenarios that students may not routinely encounter during clinical placements (Adams et al., 2024). In addition, expanding student placements to include rural and district hospitals, where diverse caseloads and systemic constraints can often closely resemble community service contexts, can help better prepare SLTs for their practical realities within the public healthcare sector. This would require coordinated support from universities, healthcare institutions and government stakeholders to increase placement capacity, provide logistical support for rural rotations and ensure access to appropriate supervision. By diversifying and enriching clinical exposure, these strategies can collectively enhance confidence and competence among newly qualified SLTs, contributing to more equitable and effective service provision in paediatric dysphagia management.

## Conclusion

The results of this study reveal the multifaceted challenges faced specifically by community service SLTs when managing children with complex medical conditions, offering unique insights into the experiences of novice clinicians at a critical stage of professional development. Understanding their perspectives is essential, as these therapists often serve in under-resourced settings with limited supervision, and their readiness and confidence directly impact the quality of paediatric dysphagia care within the public health sector. The results further underscore the importance of adequate training, resources, and MDT collaboration to support and enhance the extent to which community service SLTs feel prepared. Additionally, improving treatment outcomes for children in diverse communities will require addressing socio-economic and cultural barriers. The gaps in training and practical experience have also been found to undermine the need for ongoing professional development that guarantees that community service SLTs are prepared to effectively handle the complexities that often accompany paediatric feeding and swallowing difficulties. By targeting these challenges, we can begin to elevate the standard of care provided to these children as well as the confidence and job satisfaction of community service SLTs working within the South African public healthcare sector.

### Significance and limitations

This study is significant in its ability to help inform service delivery initiatives and curriculum reform at institutions of higher learning. The limitations of the study include: (1) use of a small and/or non-representative sample which limits the generalisation of the study findings; (2) the use of self-reported data, which allows for the possibility of a recall bias in the participants’ responses; (3) the single-case research design used restricts the ability to make broad conclusions; and (4) while cultural and linguistic barriers are discussed, the study could have explored these factors across diverse demographic groups in more detail.

### Implications and recommendations

#### Undergraduate clinical training

The findings of this study highlight the need to strengthen undergraduate clinical training in paediatric dysphagia to better prepare SLTs for independent practice, particularly within the South African public health sector. While some graduates reported feeling prepared for routine cases, many felt ill-equipped to manage medically complex presentations, largely because of inconsistent clinical exposure during training. Given their reduced practical exposure, the use of structured case-based learning and simulation is thus recommended to foster clinical reasoning, decision-making and confidence in a low-risk environment. By integrating simulation and cultural competence training, academic programmes can help to bridge the gap between theoretical knowledge and practical demands, enhancing SLTs’ readiness to provide effective, contextually appropriate care in complex paediatric settings.

#### Community service mentorship

It is recommended that some form of mentorship be provided to all SLTs in their community service year. Because there is a lack of SLTs across the country, supervision of all therapists in their community service year may not be feasible. It is therefore recommended that there be a peer support system, whereby experienced SLTs are paired with a few community service SLTs. These experienced SLTs can be in other hospitals and/or provinces.
